# A Cohort Study of Morbidity, Mortality and Health Seeking Behavior following Rural Health Center Visits by Children under 12 in Southwestern Uganda

**DOI:** 10.1371/journal.pone.0118055

**Published:** 2015-01-30

**Authors:** Matthew O. Wiens, Heng Gan, Celestine Barigye, Guohai Zhou, Elias Kumbakumba, Jerome Kabakyenga, Niranjan Kissoon, J. Mark Ansermino, Walter Karlen, Charles P. Larson, Stuart M. MacLeod

**Affiliations:** 1 School of Population and Public Health, Faculty of Medicine, University of British Columbia, Vancouver, Canada; 2 Department of Pediatrics, Mbarara University of Science and Technology, Mbarara, Uganda; 3 Guy’s & St Thomas’ Hospital, NHS Foundation Trust, London, United Kingdom; 4 Ministry of Health, Government of Uganda, Kampala, Uganda; 5 Department of Statistics, University of British Columbia, Vancouver, Canada; 6 Maternal, Newborn and Child Health Institute, Mbarara University of Science and Technology, Mbarara, Uganda; 7 Department of Pediatrics, BC Children’s Hospital and University of British Columbia, Vancouver, Canada; 8 Department of Pediatric Anesthesia, BC Children’s Hospital and University of British Columbia, Vancouver, Canada; 9 Department of Health Sciences and Technology, ETH Zurich, Zurich, Switzerland; 10 Centre for International Child Health, Child and Family Research Institute, BC Children’s Hospital and University of British Columbia, Vancouver, Canada; Centers for Disease Control and Prevention, UNITED STATES

## Abstract

**Background:**

Children discharged from hospitals in developing countries are at high risk of morbidity and mortality. However, few data describe these outcomes among children seen and discharged from rural outpatient centers.

**Objective:**

The objective of this exploratory study was to identify predictors of immediate and follow-up morbidity and mortality among children visiting a rural health center in Uganda.

**Methods:**

Subjects 0–12 years of age seeking care with a caregiver were consecutively enrolled from a single rural health center in Southwestern Uganda. Baseline variables were collected by research nurses and outcomes of referral, admission or death were recorded (immediate events). Death, hospital admission and health seeking occurring during the 30 days following the clinic visit were also determined (follow-up events). Univariate logistic regression was performed to identify baseline variables associated with immediate outcome and follow-up outcomes.

**Results:**

Over the four-month recruitment period 717 subjects were enrolled. There were 85 (11.9%) immediate events (10.1% were admitted, 2.2% were referred, none died). Forty-seven (7.8%) events occurred within 30 days after the visit (7.3% sought care from a health provider, 1.5% were admitted and 0.5% died). Variables associated with immediate events included living more than 30 minutes from the health center, age older than 5 years, having received an antimalarial prior to the visit, having seen a community health worker prior to the visit, elevated respiratory rate or temperature, and depressed weight-for-age z score or decreased oxygen saturation. These variables were not associated with follow-up events.

**Conclusions:**

Sick-child visits at a rural health center in South Western Uganda were associated with rates of mortality and subsequent admission of less than 2% in the period following the sick child visits. Other types of health seeking behavior occurred in approximately 7% of subjects during this same period. Several variables were associated with immediate events but there were no reliable predictors of follow-up events, possibly due to low statistical power.

## Background

Children who have been discharged from health facilities, especially in developing countries, are at high risk of significant morbidity and mortality. In many instances the short term mortality (weeks to months) associated with hospital discharge may be as high, or higher, than mortality during hospitalization.[1] Despite this increased vulnerability in the post-discharge period, few data exist to characterise the morbidity, mortality and health seeking behavior following primary outpatient, health center visits among rural populations in low income countries. In 2007, Lindblade et al. reported the 30 day mortality rate of 2.4% among 1711 sick children 2–60 months of age presenting to seven rural health facilities in western Kenya during a four month period.[2] Of those who died, 12% died on the same or next day and over 40% died within the first week. However, their admission status following the clinic visit was not reported limiting any conclusions on the morbidity and mortality during the period following out-patient visits. In the full cohort, the most important predictors of death included malnutrition, age less than 24 months, anemia, and severe pneumonia.

An improved understanding of the risk factors for morbidity and mortality following out-patient department visits would be of considerable utility in improving care at rural health centers. The incorporation of a simple risk score to guide admission, referral and outpatient treatment decisions may help to improve timely intervention among vulnerable children as well as reduce unnecessary intervention. The development of risk scores that can be used at rural health centers by health workers with limited training requires that risk factors, especially clinical risk factors, be measured accurately. Severe pneumonia, consistently identified as an important risk factor for post-discharge mortality, may be useful in such a risk score, but may be difficult to diagnose at rural health centers by health workers with limited training. We have an interest in using pulse oximetry as a sensitive and repeatable measure of lung injury and its role as indicator of risk while at the same time incorporating other important derived variables such as heart rate.[3] Other variables consistently noted to be associated with post-discharge mortality that should be explored in a post outpatient department (post-OPD) context include nutrition status (weight-for-age z scores) and age.

The purpose of this exploratory study was to identify predictors of both immediate and follow-up morbidity and mortality among children visiting a rural health center in Southwestern Uganda. In addition to exploring associations of variables known to be associated with post-discharge mortality, variables thought to be potentially associated with morbidity and/or mortality following out-patient department visits were also examined.

## Methods

### Study design

This was a prospective cohort study with recruitment from October2012 to January 2013, with follow-up occurring from November 2012 until May 2013.

### Population

The Kyabugimbi Health Center is level IV primary health care centre located in the Bushyenyi District of South Western Uganda at an altitude of approximately 1400 meters. In Uganda, health facilities are divided into health centers and hospitals. Health centers range from level I (lowest—village level) to level IV (highest—county level). Above the level of a health center are three levels of hospital (district, regional, and the national referral hospital). Health centers generally focus on out-patient services although many will have limited in-patient facilities, especially level IV health centers. The Kyabugimbi health center’s diagnostic capabilities are limited to those tests able to be easily performed using an optical microscope (blood smear and sputum/stool analysis), or rapid diagnostic tests (urine dipsticks, HIV, malaria). The health center has the capacity to test and refer for HIV treatment (drugs to prevent mother-to-child transmission of HIV are available). This health center can treat the common diseases of childhood including, but not limited to diarrhea, pneumonia, malaria, skin and soft tissue infections and tuberculosis. This health center conducts health promotion and immunization activities in the community. A referral strategy for these illnesses would be generally based on the national guidelines and IMCI. The catchment population of the Kyabugimbi health center IV is rural and composed primarily of households dependent upon subsistence and small-scale farming or small businesses catering to the immediate needs of the community. The nearest referral center is approximately 25 km away. In this population approximately 17% of children under-five years are considered underweight, 36% stunted and 8% wasted (unpublished data from the baseline survey conducted by Healthy Child Uganda in Bushyenyi District in 2012). The Bushenyi district has recently been the focus of a maternal-child health program, Healthy Child Uganda, funded under the Canadian government’s Muskoka Initiative. This has allowed for a substantial investment in the training of volunteer community health workers (village level health center I) and the implementation of the Integrated Community Case Management guidelines.[4] These volunteer community health workers are often the first health workers to assess children who are ill. They are trained to diagnose and treat early stages of malaria, pneumonia and diarrhea in children, as well as identify those children in need of referral to higher levels of care. The drugs available to these community health workers for the treatment of children include oral antibiotics (amoxicillin), oral anti-malarials (artemether-lumefantrine), oral rehydration salts and oral zinc. The Kyabugimbi Health Center IV offers a general service, providing pediatric, adult and maternity care. Limited in-patient pediatric facilities make this health center primarily an outpatient center. Children with more severe disease are referred to higher level health centers. The health center provides care to approximately 10–20 pediatric patients per day, with seasonal fluctuations. Most children accessing care live within 30 minutes of this clinic (either by walking or public transportation). The care is provided primarily by nurses with 1–2 years of training and clinical officers (2-year diploma trained paramedicals with diagnostic and therapeutic training). There are generally no physicians available and the centre operation is primarily during the day time. The Integrated Management of Childhood Illness (IMCI) guidelines form part of the national guidelines of care in the diagnosis and treatment of the most common childhood illnesses and is used at this center.[5]

### Eligibility

All children aged 0–12 years old presenting with a parent or guardian to the health center between October 10, 2012 and January 31, 2013 between 8am and 5pm on non-holiday weekdays were eligible for enrollment. This time period encompassed both the rainy season and early part of the dry season. Children were only enrolled on one occasion during the course of the study.

### Data collection/measurement

Following informed consent, a trained research nurse interviewed the parent/guardian of each child to collect social, demographic, health seeking, health behavior and clinical data. The research nurse obtained and recorded clinical signs including respiratory rate (tapping method)[6], blood pressure (automated), axillary temperature, and using the Phone Oximeter, [7] 1 min photoplethysmogram (PPG), blood oxygen saturation (SpO_2_) and heart rate. Anthropomorphic data (height, weight, mid-upper arm circumference) were also measured and recorded. These were recorded separately from the routine examination by the health center staff. The diagnosis made during the visit by health center staff was recorded, as was the outcome of each visit (outpatient treatment, referral, admission, death). All enrolled children received standard care during the sick-child visit. Approximately two to four months following the visit, subjects were visited at their place of residence by a field officer. During the follow-up visit the vital status (alive or dead) as well as all health seeking since the visit were recorded, as recalled by the primary caregiver of the child. The date of each health seeking episode was recorded along with the type of provider seen. PPG recordings were analyzed for artifacts and other quality degradations using Gaussian filters and cross-correlation [8] and sorted according the quality (perfect, acceptable, challenging, last resort, unusable/unavailable) and duration. This automated quality assessment was visually validated by an Anesthesiologist. Median heart rate and SpO2 were extracted from the largest artifact free PPG recording for each subject. Age-dependent demographic variables collected at enrollment were converted to age corrected z-scores according to the World Health Organization Child Growth Standards.[9] The age corrected heart rate and respiratory rate z-scores were obtained by standardizing the raw measurements using the median and SD values provided by Flemming et al.[10] The age corrected z-scores for systolic blood pressure were calculated using subjects’ height, according to the procedures previously described.[11]

### Outcomes

The primary outcomes were stratified by immediate and follow-up events. Immediate events were those occurring at the time of the initial sick child visit. These were defined (1) admission, (2) death and (3) referral. Admission was defined as an admission to the Kyabugimbi health center IV. Referral was defined as a referral made by the attending clinical officer to a higher level of care. Death was defined by death during the course of admission, or if referred, as death during transport or admission at the referral center.

Follow-up events were those events which occurred within 30 days following the initial health center visit (or following discharge/referral in those who were either admitted or referred) and were exclusive of immediate events. These were defined as a new admission to a health center/hospital, death, or visit to one of five categories of health care providers. The five categories were (1) nurse (2) doctor/clinical officer (3) community health worker (4) traditional healer and (5) untrained health worker. A secondary outcome of death and/or hospital admission within 30 days was also analyzed against potential predictor variables.

### Statistical Analysis and Sample Size

Census sampling of all children under 12 years of age attending the clinics during weekday working hours was carried out for 16 weeks. The purpose of this exploratory study was to be hypothesis generating rather than hypothesis testing. We assumed a 10% composite event rate for both immediate and follow-up events. With enrollment of 500 subjects we estimated an accrual of 50 immediate and 50 follow-up events, providing sufficient statistical power to explore possible univariate associations between potential predictor variables and these outcomes. Database preparation was done using R 3.0 (Statistical analysis was conducted in SAS 9.2 (Carey, NC)). Univariate logistic regression was used to model the association between each potential predictor variable and both immediate and follow-up events, with the odds ratio and a 95% CI being the measure of association. Since subjects could be labelled with more than one diagnosis, the analysis of diagnostic category was done as an adjusted analysis, controlling for all diagnostic categories.

### Ethical Considerations

This study received research ethics approval from the Mbarara University of Science and Technology, Uganda and the University of British Columbia, Vancouver, Canada. Written informed consent was provided by a parent or guardian of all included subjects.

## Results

**Fig 1:**
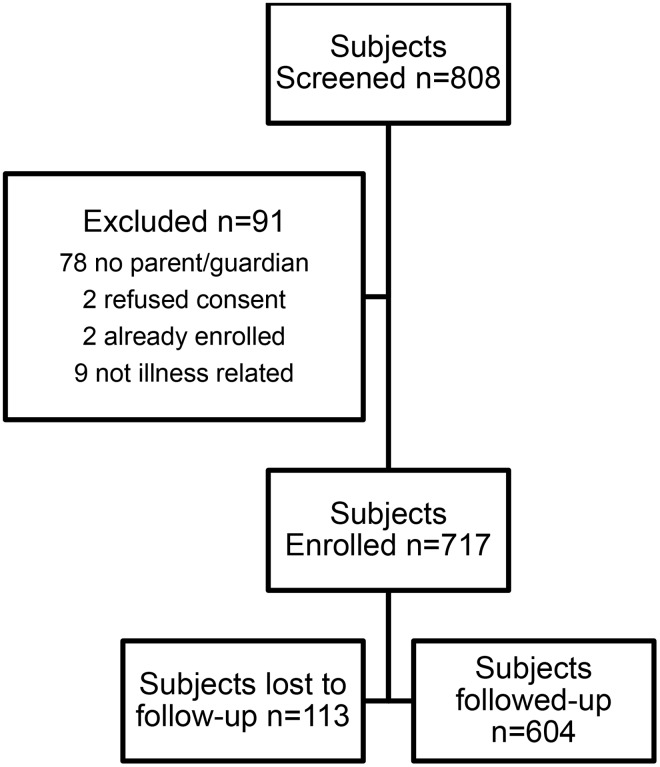
Consort Diagram of study flow.

**Fig 2:**
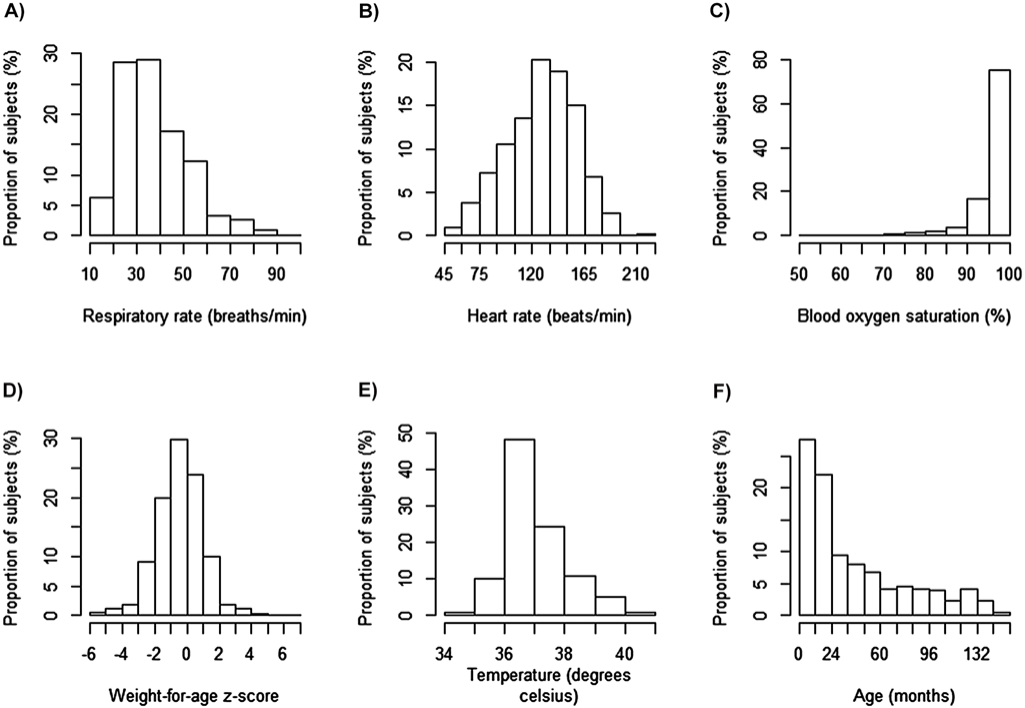
Histograms of important clinical variables collected at baseline from consented subjects.

Over the four-month recruitment period there were 808 sick-child visits, of which 717 were enrolled (89%). Of the 717 enrolled subjects, 604 (84%) received a successful follow-up visit. Of the 91 excluded subjects 78 had no parent/guardian present, 2 were already enrolled, 2 refused consent and the remaining were excluded as their visits were not due to an illness (Fig. 1). The median age of the children representing the sick child visit was 25 months (IQR 11–64) and 360 (50.2%) were male (Table 1, Fig. 2). Most subjects (75.2%) lived within 30 minutes from the health center by public transport and only 3% lived more than 60 minutes from the health center. Over 70% of subjects presented with an illness duration of less than 7 days.

**Table 1:** Baseline characteristics of study subjects.

Characteristic	Frequency/mean/median
	Total N = 717
Demographics	
Median age in months (IQR)	25 (11–64)
Male sex	360 (50.2%)
History of sibling death	20.8%
Ever breastfed	99.9%
Median number of Children in Family (IQR)	3.0 (2.0–5.0)
Mother Alive	700 (97.6%)
Maternal HIV positive	35 (4.9%)
Maternal HIV negative	575 (80.2%)
Maternal HIV unknown	107 (14.9%)
HIV status	
Known HIV positive	5 (0.7%)
Maternal education	
No education	93 (13.0%)
Less than Grade 3	78 (10.9%)
Grade 3 to Grade 7	380 (53.0%)
Some Secondary School (S1 to S6)	133 (18.6%)
Post-Secondary	33 (4.6%)
Transport cost	
≤ 1000 UGX (1000 UGX = 0.4 USD)	144 (20.1%)
1001–2000 UGX	261 (36.4%)
2001–3000 UGX	175 (24.4%)
> 3000 UGX	137 (19.1%)
Bednet use	
Never	139 (19.4%)
Sometimes	54 (7.5%)
Always	524 (73.1%)
Distance to health center (typical transport)	
< 30 min	539 (75.2%)
30–60 minutes	156 (21.8%)
> 60 minutes	22 (3.1%)
Duration of illness prior to visit	
< 7 days	524 (73.2%)
7–30 days	153 (21.4%)
> 30 days	39 (5.5%)
Seen by CHW	177 (24.7%)
Referred by CHW	134 (18.7%)
Clinical variables	
Mean RR (SD)	38.2 (14.6)
Mean HR age Z score (SD)	1.1 (0.95)
Mean RR age Z score (SD)	1.9 (2.6)
Mean SBP age Z score (SD)	0.55 (3.17)
Mean weight for age Z score (SD)	-0.44 (1.47)
Mean Temperature (SD)	37.1 (1.03)
Median SpO2 (IQR)	97.3 (95.1–98.5)

CHW: Community health worker; RR: Respiratory Rate; HR: Heart Rate; SBP: Systolic blood pressure

Diagnoses made during the sick child visits were grouped into 10 categories (Table 2). Pneumonia, clinical malaria and non-specific respiratory tract infections were the most common infection related diagnoses made, with 23%, 30% and 37% of children receiving these diagnoses, respectively. Sixty percent of subjects had a non-infectious diagnosis (eg. reactive airway disease, trauma, malnutrition etc.), although most of these were diagnosed concurrently with a suspected infection. Six hundred and six of the 717 subjects had a blood smear examined for parasitemia of which 27 (4.5%) were positive. Overall, 651 (91.4%) of children had a suspected infection and 61 (8.6%) were not diagnosed with any infectious illness.

**Table 2:** Outpatient department diagnoses.

Outpatient/Admitting diagnosis	Frequency n (%)
	Total N = 717
Respiratory tract infection (not specified)	264 (36.8%)
Malaria	221 (30.1%)
Pneumonia	164 (22.9)
Skin and Soft Tissue Infection	38 (5.3%)
Gastroenteritis	40 (5.6%)
HIV	4 (0.6%)
Tuberculosis	2 (0.3%)
Meningitis/Encephalitis	0 (0%)
Other infection	72 (10.0%)
Other non-infectious diagnosis	419 (58.4%)

### Immediate events

There were 85 (11.9%) immediate events among the 717 sick child visits. Seventy two subjects (10.1%) were admitted following assessment by the clinical officer, 16 (2.2%) were referred to a higher level of care by the clinical officer (three were admitted and referred) and no subjects died (Table 3). An immediate event occurred in 12.1% of children with suspected infections and in 6.6% of children with no suspected infection (risk difference, 5.6%; 95% CI-1.1% to 12.3%).

**Table 3:** Outcomes of subjects during SCV and during 30 day follow-up period.

Event Details	Frequency n (%)
Immediate events (Total N = 717)	
Outcome event*	85 (11.9%)
Death	0 (0%)
Immediate admission	72 (10.1%)
Referral	16 (2.2%)
30 day follow-up events (Total N = 604)	
Outcome event*	47 (7.8%)
Death during 30 day follow-up	3 (0.5%) (2 at home, 1 in hospital)
Admission post-OPD visit	8 (1.3%)
CHW visit post-OPD visit	8 (1.3%)
Physician/Clinical officer visit post-OPD visit	19 (3.2%)
Nurse visit post-OPD visit	3 (0.5%)
Traditional healer visit post-OPD visit	0 (0%)
Untrained health worker visit post-OPD visit	10 (1.7%)

* Number is lower than sum of individual events since due to event overlap

Among demographic variables distance to health center predicted immediate events, with subjects living between 30 and 60 minutes from the health center having an odds ratio of 1.95 (95% CI 1.15–3.31) when compared to those living less than 30 minutes away (Table 4). Those living greater than 60 minutes away had a non-significant odds ratio of 2.38 (95% CI 0.77–3.46); however only 3.1% of subjects were in this distance category. Neither maternal education, number of siblings nor maternal HIV status was associated with immediate events. Children older than five years had a lower odds of immediate events compared to the reference group of children under 12 months of age (OR 0.45, 95% CI 0.22–0.94). Children in the 12–24 month category and those in the 24–60 month category were not associated with higher odds of immediate events.

**Table 4:** Univariate association between baseline variables and immediate and early events.

Variable	Immediate events OR (95% CI)	Events within 30 days OR (95% CI)
	Total N = 717	Total N = 604
Demographic variables		
Male sex	1.26 (0.80–1.99)	1.69 (0.92–3.11)
Age (ref = <12months)	REF	REF
Age 12–24 months	1.15 (0.62–2.13)	1.00 (0.40–2.27)
Age 24–60 months	1.30 (0.72–2.33)	0.91 (0.40–2.06)
Age > 60 months	0.45 (0.22–0.94)	0.78 (0.34–1.77)
Transport costs (ref = ≤1k)		
1–2k	0.74 (0.37–1.45)	1.16 (0.52–2.55)
2–3k	1.15 (0.58–2.28)	0.97 (0.41–2.33)
>3k	1.79 (0.91–3.51)	0.57 (0.19–1.72)
Distance to health center (Ref = <30 min)		
30–60min	1.92 (1.16–3.17)	0.76 (0.35–1.67)
>60 min	2.70 (0.96–7.61)	0.62 (0.08–4.75)
Sibling deaths	0.53 (0.27–1.03)	0.72 (0.33–1.58)
Number of siblings (ref = ≤1)		
2–3	0.90 (0.53–1.54)	0.55 (0.25–1.21)
≥ 4	0.60 (0.34–1.06)	0.80 (0.41–1.58)
Maternal Education (ref = no education)		
Less than P3	0.95 (0.35–2.53)	0.52 (0.16–1.75)
P4–P7	1.34 (0.66–2.76)	0.51 (0.23–1.12)
≥S1	0.76 (0.33–1.80)	0.56 (0.23–1.39)
Maternal HIV status (ref = negative)		
HIV positive	1.54 (0.62–3.85)	0.89 (0.20–3.87)
HIV unknown	0.85 (0.43–1.68)	1.12 (0.48–2.60)
Health seeking/behavior variables		
Bednet use (ref = never)		
Sometimes	1.33 (0.51–3.50)	0.61 (0.07–5.62)
Always	1.24 (0.67–2.29)	2.87 (1.01–8.19)
Antibiotic prior to visit	1.03 (0.65–1.65)	1.19 (0.65–2.18)
Antimalarial prior to visit	2.17 (1.36–3.45)	0.75 (0.38–1.52)
CHW referral	1.51 (0.88–2.57)	0.81 (0.35–1.86)
Seen by CHW	2.03 (1.26–3.27)	0.70 (0.33–1.50)
Clinical variables		
Duration of illness (Ref = <7days)		
7–30 days	1.47 (0.88–2.45)	0.84 (0.39–1.80)
> 30 days	0.43 (0.10–1.81)	1.28 (0.37–4.45)
Raw RR	1.03 (1.01–1.04)	1.00 (0.98–1.02)
RR age z-score	1.09 (0.01–1.17)	1.00 (0.89–1.13)
Raw HR	1.02 (1.01–1.03)	1.00 (0.99–1.01)
HR age z-score	1.23 (1.07–1.42)	1.00 (0.83–1.19)
Raw SBP	0.98 (0.97–1.00)	1.02 (0.99–1.04)
SBP age z-score	0.96 (0.85–1.08)	1.27 (1.05–1.54)
Temperature (above 36.5) as cont. variable Per 1°C increase	2.02 (1.57–2.57)	0.68 (0.43–1.07)
Temperature (below 36.5) as cont. variable per 1°C decrease	1.13 (0.35–3.62)	2.04 (0.77–6.07)
SpO2 (per 1% decrease)	1.06 (1.02–1.09)	1.02 (0.98–1.07)
WAZ (ref = >-2)	REF	REF
WAZ-2 to WAZ-3	1.16 (0.53–2.55)	1.03 (0.35–3.00)
WAZ <-3	2.72 (1.27–5.81)	1.76 (0.59–5.28)
Diagnostic variables (adjusted*)		
Gastroenteritis	0.75 (0.17–3.40)	1.03 (0.25–4.69)
Clinical malaria	4.50 (2.44–8.30)	0.90 (0.41–1.00)
Pneumonia	4.13 (2.01–8.15)	1.80 (0.66–4.94)
Other respiratory tract infection	0.13 (0.04–0.40)	1.31 (0.46–3.70)
SSTI	0.63 (0.12–3.18)	1.69 (0.38–7.39)
Other infection	0.47 (0.13–1.64)	1.30 (0.39–4.36)
Non infection (other)	1.58 (0.69–3.62)	0.99 (0.30–3.28)

WAZ: Weight for age z-score; SSTI: Skin and soft tissue infection

*adjusted for other diagnoses due to overlapping diagnoses

Upon analysis of health seeking and behavior variables, having received an antimalarial prior to the visit was highly predictive of an immediate event with an odds ratio of 2.17 (95% CI 1.36–3.45). A total of 202 (28%) of subjects had used an antimalarial prior to the sick-child visit. Antimalarial use prior to the health visit was associated with being seen (or referred) by a community health worker (p<0.0001) with 60% of those referred by their community health worker having received an antimalarial versus 20% of those not referred. Antibiotic use prior to the sick-child visit occurred in 265 (37%) of subjects, but was not associated with immediate events despite a similar distribution of antibiotics between those referred/seen by their community health worker and those not seen or referred (p<0.0001). Having been seen by a community health worker for the presenting illness predicted an immediate outcome (OR 2.03, 95% CI 1.26–3.27), while being referred by the community health worker was not associated with this event (OR 1.51, 95% CI 0.88–2.57).

Elevated raw and age adjusted respiratory rates and heart rate were associated with immediate events. Elevation in temperature was highly significant, with each degree increase above 36.5 C being associated with an odds ratio of 2.02 (95% CI 1.57–2.57) of immediate events. SpO2, analysed as a continuous variable, was associated with an immediate event. For each 1% decrease in SpO2 there was a corresponding 6% increase in the odds of immediate events.

Children who were severely underweight (defined as weight for age z scores less than-3) had a higher odds of immediate events with an odds ratio of 2.72 (95% CI 1.27–5.81). Children who were moderately underweight (defined as a weight for age z-score between-2 and-3) did not have a statistically significantly higher odds of immediate events.

An adjusted analysis of the diagnostic categories was made to determine their association with immediate events. Both clinical malaria and pneumonia were highly associated with immediate events with statistically significant odds ratios of 4.50 and 4.13, respectively. Non-specific respiratory tract infections had a strong negative association with immediate events (OR 0.13, 95% CI 0.04–0.40).

### Follow-up Events

Forty-seven (7.8%) events occurred during the first 30 days after the initial sick child visit in the 604 subjects who were successfully followed-up (Table 3). During this time 3 (0.5%) children died, 8 (1.3%) were admitted to a higher level of care facility and 44 (7.3%) were taken to one of 5 categories of health care providers. Among the 44 children seen by a health care provider, 19 (44%) were seen by a physician/clinical officer, 10 (23%) by an untrained health worker, eight by a community health worker (18%), 3 (6.8%) by a nurse and none by a traditional healer. The variables associated with early events were not associated with late events. In the demographics and health behavior category the consistent use of a bed nets was paradoxically associated with a higher odds of follow-up events when compared to not using a bed net. In the clinical category only a higher age adjusted z-score for systolic blood pressure was associated with higher odds of the event.

The secondary outcome of admission and/or death within 30 days occurred in 11 study subjects. Analysis of potential predictor variables against this outcome found that a low SpO2, analyzed as a continuous variable, was significantly associated with this event (OR 1.07, 95% CI 1.01–1.14) with each percent reduction in SpO2 being associated with an approximate 7% increase in the odds of either death or subsequent admission.

One hundred and thirteen subjects were lost to follow-up (16%). Children who were successfully followed-up were similar to those who were lost to follow-up, although some differences were noted. Statistically significant differences were present in maternal education, number of siblings, baseline respiratory rate and baseline oxygen saturation (Table 5).

**Table 5:** Comparison of baseline characteristics of those followed-up and those lost to follow-up.

Characteristic	Followed-up Frequency/mean/median	Lost to follow-up Frequency/mean/median	P-value
	Total N = 604	Total N = 113	
Demographics			
Median age (IQR)	25 (12–68)	23 (10–48)	0.10
Male sex	49.8	52.2	0.6
Sibling death	21.7	15.9	0.16
Med. children in family (IQR)	3 (2–5)	3 (2–4)	0.03
Mother alive	2.3	2.6	0.80
Transport			
Maternal HIV status			0.058
Maternal HIV+	4.8	5.3	
Maternal HIV-	81.6	72.6	
Maternal HIV unknown	13.6	22.1	
Maternal education			0.05
No education	13.1	12.4	
Less than Grade 3	9.4	18.6	
Grade 3—Grade 7	53.3	51.3	
Secondary School (S1–S6)	19.5	13.3	
Post-Secondary	4.6	4.4	
Transport costs			0.058
≤ 1000 UGX	20.7	16.8	
1001–2000 UGX	36.3	37.2	
2001–3000 UGX	25.5	18.6	
> 3000 UGX	17.6	27.4	
Bednet use			0.72
Never	18.9	22.1	
Sometimes	7.6	7.1	
Always	73.5	70.8	
Distance from health center			0.28
< 30 min	76.2	69.9	
30–60 minutes	20.7	27.4	
> 60 minutes	3.15	2.7	
Duration of illness prior to visit			0.30
< 7 days	73.0	74.3	
7–30 days	22.1	17.7	
> 30 days	5.0	8.0	
Clinical variables			
Mean RR (SD)	37.6 (14.7)	41.3 (13.8)	0.01
Mean RR age z-score (SD)	2.3 (3.1)	1.8 (2.5)	0.05
Mean HR age z-score (SD)	1.1 (1.7)	1.3 (1.6)	0.36
Mean SBP age z-score (SD)	0.5 (1.4)	1.1 (7.3)	0.06
Mean WAZ (SD)	-0.45 (1.5)	-0.40 (1.6)	0.80
Mean temperature (SD)	37.1 (1.0)	37.2 (1.0)	0.15
Median SpO2	97.5 (95.3–98.6)	96.4 (94.0–98.2)	0.007

## Discussion

Young age, distance from health facility, previous contact with a community health worker as well as several vital signs were associated with admission or referral following sick-child visits at a rural health center in South Western Uganda, but were not associated with follow-up events including death, subsequent admission or further health seeking behavior. Health centers, rather than hospitals, are often the point of entry for sick children, especially rural children, in sub-Saharan Africa. It is therefore important to understand the epidemiology of sick-child visits in this context. In this exploratory study, children visiting a rural level IV health center for care were enrolled and outcomes were assessed immediately and at one month following their visit.

A recent systematic review of post-discharge morbidity and mortality found that children entering the post-discharge period are vulnerable, often at a higher risk of mortality than during the hospitalization period.[1] While this observation highlights the vulnerability of hospitalized children in resource poor countries, far more children are assessed (primarily as outpatients) at community health centers than at referral centers when ill. These health centers may be far from referral centers and be handicapped by lower staff ratios, less expertise and training, less equipment and less medication. The ability to identify children who remain vulnerable following rural health center visits would be of substantial utility in designing community health interventions which could be applied during health visits. The development of risk-scoring tools that use simple and easily measured variables to predict vulnerability could significantly aid such efforts.

The findings of this study suggest that future studies aiming to link illness related variables to morbidity and mortality following sick-child visits should be designed to capture more clinically significant outcomes, such as death and admission. The frequency of such outcomes (about 2%) in this study partly reflect the difficulty of conducting such studies at the health center level and may be an important reason why so little data currently exist in this area. We estimate that a study of approximately 5000 subjects would be necessary to accrue the recommended 100 events to be able to reliably attempt the development of a 10-variable prediction tool.[12] While conducting a prospective study to create a clinical prediction model may therefore be more feasible at the hospital level, where the level of illness and risk post-discharge morbidity and mortality are much higher, such a tool may not have sufficient external validity to be applied at the health center level.

Geographic barriers to health care in rural areas have been well described.[13,14] In this study distance was measured by self-reported travel time and self-reported travel costs. In the analysis, transport time but not transport cost, was associated with immediate events. Neither cost nor time was associated with follow-up events. A recent study from rural South Western Uganda found that distance as measured by self-reported time or cost was not associated with missed HIV clinic visits, but that distance as measured by straight-line GPS was highly associated with missed clinic visits.[15] Future studies examining morbidity, mortality and health seeking among pediatric populations should consider using more objective means of distance measurement, such as GPS tracking, rather than self-report.

Prior consultation with a community health worker, but not referral by a community health worker, was associated with immediate events. This suggests that those seen but not referred were more vulnerable than the other enrolled children at the time of the sick child visit compared to those who were referred. No temporal data was collected on the timing of these visits, but it may be the case that those who were not referred were ineffectively treated in the community and therefore had delayed transfer to a higher level of care. Also, those with antimalarial treatment prior to the sick child visit had a higher probability of admission or referral, indicating that perhaps an incorrect malaria diagnosis or inadequate malaria treatment in the community was possible. The fact that most of those who had been either seen or referred by a community health worker had prior exposure to antibiotics or antimalarials suggests that the community health workers commonly provided or recommended the use of these drugs. More research on referral patterns, treatment strategies and outcomes following community health worker visits is required to further understand these observations.

The association between clinical variables and immediate events is not surprising since these variables are closely tied to the decision algorithms used in determining the need for admission and referral. The diagnosis of malaria or pneumonia by the clinic workers was also associated with immediate events, but not follow-up events. This is in contrast to several studies of post hospitalization which clearly show that diagnoses like pneumonia and low weight-for-age Z scores are associated with post-discharge morbidity and mortality.[16,17] Our study, however, was comprised mostly of children who were not admitted and the outcomes of admission and subsequent health-seeking were less robust than outcomes of re-admission and death often used in post-discharge research.

There were too few deaths to conduct any conclusive analyses using this outcome alone. However, a secondary analysis limiting the outcome of interest to death or admission within 30 days (n = 11) was not associated with any of the prospectively collected variables with the exception of low SpO2. In this analysis, for each 1% decrease in SpO2 the odds of the negative outcome increased by approximately 7%. The altitude of approximately 1400 meters at the health center resulted in a relatively low median (97.3%) oxygen saturation and would increase the statistical power in identifying children with impaired gas exchange (steep part of the oxygen dissociation curve). Further study on the utility of oxygen saturation as a predictor of post sick child visits is required and this result should be interpreted cautiously since this was a secondary analysis.

This study is subject to several limitations. First, and most importantly, the clinical variables collected for the study during the sick child visit were available for the health care center staff seeing the child. Therefore, although the research nurses did not determine or implement a treatment plan, the additional variables collected may have influenced the decision to admit or refer the subject.

Loss to follow-up was a factor in approximately 16% of enrolled subjects. Those who were lost to follow-up may have been more vulnerable than those who were found and interviewed, limiting our ability to detect important predictors of health seeking following admission. While most characteristics were similar between those found and those lost to follow-up, differences in baseline respiratory rate, oxygen saturation, maternal education and the number of siblings suggest that those lost to follow-up may have been more vulnerable. The limited sample size and the short period over which this data was collected contributed to both the possibility of type II error as well as limited insight into the seasonal variability of illness and its effect on illness and health seeking following the initial sick child visit. The duration of time between discharge/release from the health center following the sick-child visit and the time of data collection may have led to recall bias. It is possible that mothers were more likely to recall more significant episodes of illness/care seeking and that less significant episodes were missed. Finally, our post-visit outcome combined three different events: death, admission and further health care seeking. Clearly these are very different types of events and it is likely that many instances of health care seeking were for relatively benign conditions and likely to be unrelated to the initial sick child visit. This additional noise in the outcome will significantly limit detection of important parameters present at the visit that are associated with important post-visit illness.

In conclusion, this study showed that sick-child visits at a rural health center in South Western Uganda were associated with rates of mortality and subsequent admission of less than 2% and rates of health seeking of 7% in the 30-day period following the sick child visits. Age, distance to health facility, diagnosis and several clinical variables were associated with immediate events but there were no reliable predictors of 30-day follow-up events identified in this study.
